# Associations Among Depressive Symptoms, Childhood Abuse, Neuroticism, Social Support, and Coping Style in the Population Covering General Adults, Depressed Patients, Bipolar Disorder Patients, and High Risk Population for Depression

**DOI:** 10.3389/fpsyg.2019.01321

**Published:** 2019-06-05

**Authors:** Jia Zhou, Lei Feng, Changqing Hu, Christine Pao, Le Xiao, Gang Wang

**Affiliations:** ^1^The National Clinical Research Center for Mental Disorders & Beijing Key Laboratory of Mental Disorders, Beijing Anding Hospital, Capital Medical University, Beijing, China; ^2^Advanced Innovation Center for Human Brain Protection, Capital Medical University, Beijing, China; ^3^Department of Psychiatry, School of Medicine, University of North Carolina at Chapel Hill, Chapel Hill, NC, United States

**Keywords:** childhood abuse, depression, neuroticism, social support, coping style

## Abstract

**Background:**

Exposure to childhood abuse has been identified as a salient risk factor for the development of depression. However, the mediating factors between childhood abuse and depressive symptoms have not been sufficiently elucidated. This study aims to investigate the mediating effects of neuroticism, social support, and coping style between childhood abuse and depressive symptoms in population covering general adults, depressed patients, bipolar disorder patients, and high risk population for depression.

**Methods:**

This is a cross-sectional study. Five validated questionnaires were used to measure the psychological outcomes (Childhood Trauma Questionnaire CTQ-SF, Eysenck Personality Questionnaire EPQR-S, Social Support Rating Scale SSRS, Simplified Coping Style Questionnaire SCSQ, and Patient Health Questionnaire-9 PHQ-9) of 312 participants. Multiple regressions and structural equation modeling (SEM) were used to conduct data analysis.

**Results:**

Multiple regression analysis and SEM showed a significant association between childhood emotional abuse and depression symptoms. Neuroticism, use of social support, and active coping style were important mediating variables of this association. The *R*^2^ for our model was 0.456, indicating that 45.6% of the variability in depressive symptoms can be explained by the model.

**Conclusion:**

This study suggested that neuroticism, active coping, and use of social support play important role in mediating the effects of childhood abuse on adult depressive symptoms.

## Introduction

Both the high prevalence and heavy burden of mental disorders have been recognized worldwide. Childhood abuse is a major public health problem that has immediate adverse impacts and long-term negative effects on mental health (McCabe et al., 2018; Xie et al., 2018). Robust associations have been documented for retrospectively reported childhood maltreatment and adult mental disorders in numerous studies. For example, recent studies pointed out that 33% of the risk for psychosis and 22.9% of the risk for mood disorders can be attributable to childhood maltreatment (Kessler et al., 2010; Varese et al., 2012; DeRosse et al., 2014). The nationally representative epidemiological surveys conducted by World Health Organization (WHO) indicated that 38.8% of the respondents reported some form of childhood adversity (Kessler et al., 2010). The result of another epidemiological study suggested that in a predictive sense, childhood adversities explain the incidence of up to 32.4% of all psychiatric disorders in adulthood (Green et al., 2010). Substantial evidence from meta-analyses both of cross-sectional and prospective studies also found that childhood abuse was strongly associated with the development of depressive symptoms in adulthood (Lindert et al., 2014; Infurna et al., 2016). However, the mediating factors between childhood abuse and depressive symptoms have not been sufficiently elucidated. Therefore, identifying etiology processes involved in this pathway may contribute to finding effective strategies for depression prevention (Scheuer et al., 2018).

Personality is considered to be a major factor in determining psychological wellbeing (McHugh and Lawlor, 2012; Mannino et al., 2015; Granieri et al., 2017; Sideli et al., 2018). The empirical evidence that childhood maltreatment increases the risk for developing maladaptive personality traits has been documented in previous research (Rogosch and Cicchetti, 2004; Hengartner et al., 2015). The heterogeneous developmental trajectories of personality patterns during childhood have been proposed as an underlying mechanism to explain the relationship of childhood abuse with psychopathology in adult (Kim et al., 2009). Both in MDD patients and in nonclinical general adult population, the mediator effect of personality traits on the association between childhood abuse and depressive symptoms severity has been discovered with SEM (Nakai et al., 2014; Toda et al., 2016). Researches have shown that childhood maltreatment was consistently associated with high neuroticism (Moran et al., 2011; Mc Elroy and Hevey, 2014; Hengartner et al., 2015; Hovens et al., 2016). Neuroticism, one of the major temperamental basic personality traits, implies negative affectivity or negative emotionality (Watson et al., 2005). It is listed as a well-established risk factor for the onset of MDD in the DSM, Fifth Edition (DSM-5) (Robinson et al., 2014). Neuroticism, indicating a tendency to have unrealistic ideas, an inability to control urges, and inefficient ways of coping with stress, attributes to an increased risk of affective disorders (Ormel and Wohlfarth, 1991; Spinhoven et al., 2010). Many studies suggested that personality characteristics, especially neuroticism (Kendler and Gardner, 2011), appear to be a mediating factor in the relationship between childhood abuse and major depression (Nakai et al., 2014; Schulz et al., 2017).

Social support involves affection and warmth, which helps individuals with building resilience and coping with adverse circumstances effectively. By offering feelings of being accompanied and greater immunity toward negative mental health outcomes associated with childhood maltreatment (Reinelt et al., 2015), social support can play an important role in protecting against mental disorders (Sperry and Widom, 2013; Lagdon et al., 2018). For example, Lagdon et al. (2018) reported that individuals who perceived greater social support were significantly less likely to develop depression in a group of university students who had experienced childhood maltreatment. Similarly, Seeds et al. (2010) investigated the mediating role of social support between childhood maltreatment and adolescent depression and found that adolescents who experienced abuse might feel that they were isolated from their support system and others would be unavailable when they need assistance. In addition, it has been evidenced from a prospective cohort design study that social support plays a significant role in mediating and moderating the relationship between childhood abuse and subsequent outcomes (Sperry and Widom, 2013). On the other hand, childhood maltreatment was associated with an increased risk of perceived social isolation due to low self-esteem and distrust of others (Spinazzola et al., 2005; Sheikh, 2018). Previous researches have documented that it is difficult for survivors of childhood maltreatment to establish secure attachments to others (Pepin and Banyard, 2006; Whisman, 2006). Children with abuse histories are more aggressive and less intimate when interacting with others (Haskett and Kistner, 1991; Bolger et al., 1998). All this in turn will result in lower perceived social support from friends and family (Colman and Widom, 2004; Kim and Cicchetti, 2010).

The level of social support acts as the possible mediator of personality and depression (Gullo et al., 2015; Mannino et al., 2015). It is possible that social support affect the extent to which personality dimensions networks affect mental health outcomes. Due to different socialized tendencies, individuals with high neuroticism tend to perceive and elicit lower social support to keep mental wellbeing compared with people who are high in extroversion (McHugh and Lawlor, 2012). The studies of Kendler found that high levels of neuroticism also predicted low levels of social support and high risk of major depression (Kendler et al., 2002, 2006). The study of Finch and Graziano (2001) found that perceived satisfaction with social support and social exchanges are playing mediating role of the association between neuroticism and depression. This is because neuroticism influences the formation and maintenance of supportive social relationships and interferes with the reception of social support (Lahey, 2009). Social support is proved to be associated with depression and mediates the relationship between neuroticism and depression (Finch and Graziano, 2001).

Perceived social support is a classical coping resource, which appears to influence coping strategies adopted by people under stress. Another study also argued that active coping strategies are conducive to positive psychosocial outcomes (Hill et al., 1995). Positive coping style was negatively associated with depression (Falgares et al., 2019). As mentioned earlier, adults who reported childhood abuse may have less ability or opportunity to seek support, and this implies that they are less likely to enact positive coping methods when dealing with adverse events (Kong and Moorman, 2015). Lazarus and Folkman (1984) proposed social support–stress–coping paradigm hypothetical model, which has been previously cited as a conceptual framework to explore the relationship among social support, coping style, and drug abuse (Nyamathi et al., 1995). Previous studies emphasized coping strategy’s mediating role between social support and individuals’ adjustment outcomes like depression (Fleishman et al., 2000; Rayburn et al., 2005). Individuals who have adequate social support will be more inclined to use positive coping strategies because they believe their social relationship could give full support and their coping efforts are more effective (Dunkel-Schetter et al., 1987).

**FIGURE 1:**
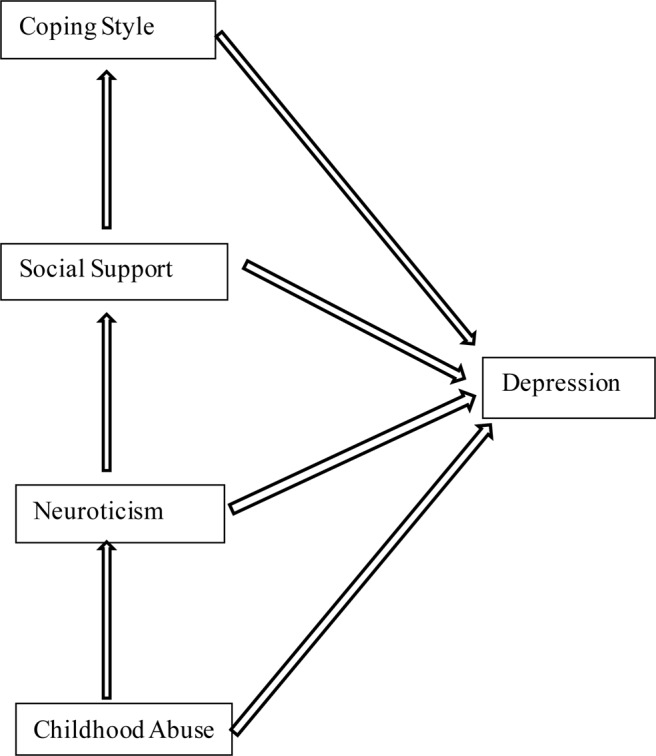
The hypothesis. SEM of the hypothesis of this study. In this model, childhood abuse, neuroticism, social support, and coping style predict depressive symptoms.

Therefore, we thought it was important to analyze the interactions among the five factors: childhood abuse, neuroticism, social support, coping style, and depressive symptoms. Previous studies have documented that the effects of childhood abuse on depressive symptoms and the mediating factors between them were analogous both in MDD patients and general adults (Enns et al., 2000; Nakai et al., 2014, 2015; Toda et al., 2016; Ono Y. et al., 2017). No study has ever included all the four risk factors in one path analysis to detect the influence order and calculate the total effect of them on the depressive symptoms. This research aims to establish a SEM and examine the direct and indirect relationships among childhood abuse, neuroticism personality, social support, coping strategies, and depression symptoms in the population covering general adults, depressed patients, bipolar disorder patients, and high risk population for depression. SEM can provide theory-based models for understanding complicated relationships between multiple factors and further clarify the etiology of depressive symptoms affected or caused by childhood maltreatment. Based on previous findings from the literatures, we build a theoretical model (Figure 1) and hypothesized that childhood abuse affects depressive symptoms through neuroticism, social support, and coping style in turn.

## Materials and Methods

### Sample

This study was conducted between January 2014 and December 2017. Depressed patients and bipolar disorder patients were recruited from outpatient clinic of Beijing Anding Hospital. Beijing Anding Hospital is a university-affiliated teaching hospital in China that serves a population of approximately 19 million people and has 1100 outpatient visits daily. Volunteers for general adults and high risk population for depression were recruited through advertisement in communities and the hospital.

Patients with depression were eligible for the study if they were: (1) male or female aged 18–55 years, inpatient or outpatient; (2) met DSM-IV diagnostic criteria for depression and ascertained by the Chinese version of the MINI Version 5.0 modules on major depression; (3) not being treated with any psychiatric medications (e.g., antidepressants) in the last 2 weeks; (4) primary school education level or above; (5) patient or legal guardian signed the informed consent; and (6) course of disease is more than 8 years, experienced at least three depressive episodes, with no history of mania or hypomania. The exclusion criteria for patients with depression were: (1) current or previous diagnosis of other psychiatric disorders (e.g., schizophrenia); (2) depressive disorder secondary to organic etiology; (3) severe and unstable physical disease leading to inability to complete the questionnaire survey and assessment; (4) history of alcohol or psychoactive substance abuse within the last year; (5) pregnancy, lactation, or use of contraceptive drugs; (6) current serious suicidal thoughts; (7) epilepsy and other organic brain disorders; and (8) treatment with twitch or magnetic stimulation treatment in the past 3 months.

Volunteers for general adults were eligible for the study if they were: (1) male or female aged 18–55 years, (2) no current or previous mental disorder diagnoses, (3) primary school education level or above, (4) normal intelligence (i.e., no diagnosis of intellectual disability), and (5) signed the informed consent. The exclusion criteria for the general adults were: (1) chronic physical disease; (2) long-term use of painkillers; and (3) pregnancy, lactation, or use of contraceptive drugs.

Bipolar disorder patients were eligible for the study if they were: (1) male or female aged 18–55 years, inpatient or outpatient; (2) primary school education level or above; (3) met DSM-IV diagnostic criteria for bipolar disorder and ascertained by the Chinese version of the MINI Version 5.0 modules on bipolar disorder; (4) not treated with any psychiatric medications in the last 2 weeks; and (5) patients or their legal guardian signed the informed consent. The exclusion criteria for the bipolar disorder patients were: (1) current or previous diagnosis of other psychiatric disorders (e.g., schizophrenia); (2) bipolar disorder secondary to organic etiology; (3) severe and unstable physical disease leading to inability to complete the questionnaire survey and assessment; (4) history of alcohol or psychoactive substance abuse within the past year; (5) pregnancy, lactation, or use of contraceptive drugs; (6) current serious suicidal thoughts; (7) epilepsy and other organic brain disorders; and (8) treatment with twitch or magnetic stimulation treatment in the past 3 months.

High risk population for depression was eligible for the study if they were: (1) male or female aged 18–55 years, inpatient or outpatient; (2) did not meet DSM-IV diagnostic criteria for MDD; (3) depressive mood and at least one of the other eight symptoms of depression; (4) categorized in at least one of three high risk groups including (4a) adults whose children are diagnosed of mental dysfunction, autism spectrum disorder, or physical disability for more than 1 year [parents of children with severe mental illness or physical disability were at greater risk of having depression (Xiong et al., 2011; Cohrs and Leslie, 2017)], (4b) adults who were clearly diagnosed with hypertension, diabetes, chronic pain, or cancer in the past month [comorbid severe chronic illness and depression is a gradually recognized clinical problem (Holt et al., 2014; Graham and Smith, 2016)], or (4c) medical staff working in the emergency department for the past 3 years [studies indicated that staff members working in emergency units are in high risk of depression and anxiety (Bennett, 2004; Erdur, 2006; Katz et al., 2006)]; (5) primary school education level or above; and (6) signed the informed consent. The exclusion criteria for the high risk population for depression were: (1) current or previous diagnosis of other psychiatric disorders (e.g., schizophrenia); (2) severe and unstable physical disease leading to inability to complete the questionnaire survey and assessment; (3) epilepsy and other organic brain disorders; and (4) pregnancy, lactation, or use of contraceptive drugs.

Ethical approval was obtained from the Ethics Review Committee of the studied hospital. All patients participated on a voluntary basis and gave their written informed consent before data collection.

### Measures

#### Socio-Demographic and Clinical Measures

Relevant socio-demographic characteristics including age, gender, employment status, marital status, presence of children, physical disease, FMDR, and SMDR are considered in the current study.

#### Childhood Trauma Questionnaire-Short Form (CTQ-SF)

The CTQ-SF consisting of 28 items is a self-report measure developed by Bernstein et al. (1994, 2003). It is adopted to measure the severity of five different types of childhood adversity: EA, SA, PA, EN, and PN. Each subscale is composed of five items and three items are included as a validity scale. The response choices for each item are on a five-point Likert-type scale ranging from 1 = *never true* to 5 = *very often true*, plus. The Chinese version has good validity and reliability performed by Zhang (2011).

#### Eysenck Personality Questionnaire-Revised Short Form (EPQR-S)

Eysenck Personality Questionnaire-Revised Short Form, developed by Eysenck et al. (1985), aims to assess four personality dimensions: extroversion or introversion, psychoticism, lying and dissimulation, and neuroticism. It is comprised of 48 questions, evaluated by the participants with an “agreement” or “disagreement” answers; and the scores of each dimension range from 0 to 12. This study focused on the neuroticism score assessed by the neuroticism subscale (12 items) of EPQR-S, following the method of Kendler et al. (2004). Neuroticism score reflects how anxious, worried, moody, and frequently depressed the subject is. EPQR-S has been widely used in China and has been validated (Qian, 2000).

#### The Social Support Rating Scale (SSRS)

The SSRS consisted of 10 items. It evaluates three types of social support: objective support, subjective support, and use of social support. Objective social support assesses the extent of practical support that the individual received from the social network. Subjective support is the individual’s level of satisfaction with being respected, supported, and understood by others in their interpersonal environment. Use of social support reflects the individual’s active use of available social supports (Cheng et al., 2008). Higher scores indicate better social support from family, friends, and others. The SSRS has been used in many other studies and proved to have high reliability and validity (Cheng et al., 2008; Gao et al., 2009; Xie et al., 2009; Xu and Wei, 2013; Wang et al., 2015).

#### Simplified Coping Style Questionnaire (SCSQ)

Simplified Coping Style Questionnaire is a 20-item self-report questionnaire focusing on reflecting participants’ coping tendencies (“active coping” and “passive coping”). Active coping covers the first 12 items and emphasizes the characteristics of positive or active coping. Passive coping covers the remaining eight items and focuses on the traits of negative coping. A four-point Likert scale is used for all items, ranging from 0 to 3 (0 = *never*, 1 = *seldom*, 2 = *often*, 3 = *always*). The instrument was first developed by Xie (1998) and it has adequate reliability (Li et al., 2014a).

#### Patient Health Questionnaire-9 (PHQ-9)

The PHQ-9 is a widely used self-reported scale to assess the severity of depressive symptoms in the previous 2 weeks. It has nine items reflecting all nine symptom criteria for MDD as described in DSM-IV (Kroenke et al., 2001). Each item is scored on a four-point Likert scale as 0 = *not at all*, 1 = *several days*, 2 = *more than half the days*, 3 = *nearly every day*, with a total score ranging from 0 to 27. Higher score means more serious depressive symptoms. The Chinese version of the PHQ-9 has been validated in Chinese samples with substantial internal consistency (Cronbach’s α = 0.89) and well-established psychometric properties (Chen et al., 2013).

### Data Analysis

We took three steps to verify our hypothesis that childhood abuse affects depressive symptoms through neuroticism, social support, and coping style in turn. Firstly, we examined the differences in PHQ-9 score between different demographic characteristics using the Mann–Whitney test. Next, Spearman’s rank correlation coefficient and multiple regression analysis for CTQ-SF, EPQR-S, SSRS, SCSQ, and PHQ-9 were computed in SAS9.4 with two-tailed probability value of <0.05 considered to be statistically significant. Finally, we used an SEM approach by AMOS17.0 (IBM Corp., Chicago, IL, United States) to test the theoretical model (Figure 1) relating childhood abuse, neuroticism, social support, coping style, and depressive symptoms.

An SEM was designed based on the hypothesis. In this path analysis, the direct and indirect effects were analyzed using maximum-likelihood covariance estimation. We calculated the indices of goodness of fit to assess the statistical evaluation of the SEM, and a GFI value above 0.90 indicates a good fit. The standardized coefficients were shown.

## Results

### Demographic Characteristics, CTQ-SF, EPQR-S, SSRS, SCSQ, and PHQ-9 of the Participants

A total of 371 participants were recruited for this study and 59 participants did not complete the questionnaire or had missing key indicators data. 312 participants with complete data were included for data analysis, including 145 depressed patients, 21 bipolar disorder patients, 45 high risk people for depression, and 101 general adults. The mean age was 34.76 ± 10.90 years. There were 130 male (41.67%) and 182 female (58.33%).

We showed the demographic characteristics and the relationships between CTQ-SF, EPQR-S, SSRS, SCSQ, and PHQ-9, respectively, in Table 1. In the effective sample of 312 participants, unemployed status was associated with more severe depressive symptoms (PHQ-9). EA, EN, PN, passive coping scores of SCSQ, and the neuroticism scores were significantly and positively correlated with PHQ-9 score. Three dimensions of SSRS and active coping scores of SCSQ were significantly and negatively correlated with PHQ-9 score.

**Table 1:** Characteristics, CTQ-SF, EPQR-S, SSRS, SCSQ, and correlation with PHQ-9 or effects on PHQ-9 in the participants.

Characteristics or measures	Value (number or mean ±*SD*)	Correlation with PHQ (ρ) or effect on PHQ-9 (mean ±*SD* of PHQ-9 scores, Mann–Whitney test)
Gender (male:female)	130:182	Male 9.22 ± 7.28 vs. female 10.19 ± 8.40, *df* = 1, *Z* = -0.67, *P* = 0.504 (Mann–Whitney test)
Age	34.76 ± 10.90	ρ = -0.078, *P* = 0.167 (Spearman’s correlation)
Living-alone (yes:no)	52:260	Yes 10.17 ± 8.11 vs. no 9.70 ± 7.94, *df* = 1, *Z* = 0.43, *P* = 0.671 (Mann–Whitney test)
Offspring (yes:no)	167:145	Yes 14.53 ± 6.83 vs. no 15.11 ± 6.58, *df* = 1, *Z* = -1.74, *P* = 0.082 (Mann–Whitney test)
Comorbidity of physical disease (yes:no)	116:196	Yes 9.54 ± 7.37 vs. no 9.92 ± 8.30, *df* = 1, *Z* = 0.009, *P* = 0.993 (Mann–Whitney test)
Employment status (employed:unemployed)	267:45	Employed 9.14 ± 7.72 vs. unemployed 13.58 ± 8.35, *df* = 1, *Z* = 3.34, *P* = 0.0008^∗∗^ (Mann–Whitney test)
FMDR (yes:no)	31:181	Yes 10.39 ± 8.58 vs. no 9.72 ± 7.90, *df* = 1, *Z* = 0.28, *P* = 0.782 (Mann–Whitney test)
SMDR (yes:no)	21:291	Yes 11.76 ± 9.07 vs. no 9.64 ± 7.87, *df* = 1, *Z* = 0.97, *P* = 0.331 (Mann–Whitney test)
Marital status (married:unmarried)	201:111	Yes 10.29 ± 7.91 vs. no 8.86 ± 8.00, *df* = 1, *Z* = -1.56, *P* = 0.118 (Mann–Whitney test)
PHQ-9 score	15.29 ± 6.50	
CTQ-SF		
SA	5.39 ± 1.24	ρ = -0.059, *P* = 0.296 (Spearman’s correlation)
PA	5.63 ± 1.78	ρ = -0.097, *P* = 0.087 (Spearman’s correlation)
EA	6.63 ± 2.90	ρ = 0.297, *P* < 0.001^∗∗^ (Spearman’s correlation)
EN	9.81 ± 4.42	ρ = 0.335, *P* < 0.001^∗∗^ (Spearman’s correlation)
PN	7.50 ± 2.65	ρ = 0.279, *P* < 0.001^∗∗^ (Spearman’s correlation)
Neuroticism score of EPQR-S	6.41 ± 4.07	ρ = 0.676, *P* < 0.001^∗∗^ (Spearman’s correlation)
SSRS		
Objective support	18.65 ± 4.19	ρ = -0.229, *P* < 0.001^∗∗^ (Spearman’s correlation)
Subjective support	8.90 ± 2.93	ρ = -0.198, *P* < 0.001^∗∗^ (Spearman’s correlation)
Use of social support	7.13 ± 2.05	ρ = -0.363, *P* < 0.001^∗∗^ (Spearman’s correlation)
SCSQ		
Active coping	19.46 ± 6.97	ρ = -0.463, *P* < 0.001^∗∗^ (Spearman’s correlation)
Passive coping	8.83 ± 4.10	ρ = 0.138, *P* = 0.015^∗^ (Spearman’s correlation)

*Data presented as mean ±SD or number. ρ = Spearman’s rank correlation coefficient. ^∗^P < 0.05. ^∗∗^P < 0.01. CTQ-SF, The Childhood Trauma Questionnaire; EPQR-S, Eysenck Personality Questionnaire; SSRS, The Social Support Rating Scale; SCSQ, Simplified Coping Style Questionnaire; PHQ-9, Patient Health Questionnaire-9; SD, standard deviation; df, the degrees of freedom.*

#### Stepwise Multiple Regression Analysis of the Explanatory Variables on the PHQ-9

To find out the predictor of the severity of depressive symptoms, stepwise multiple regression analysis was performed and the result was shown in Table 2. Twenty potential factors were retained in the multiple linear regression analysis as listed in Table 1. In the final model, a higher level of EA and neuroticism was intimately associated with a higher level of depression. Active coping and use of social support were found to be negatively associated with depression. Multi-collinearity was denied in the multiple regression analysis. The *R*^2^ for our model was 0.456, indicating that 45.6% of the variability in depressive symptoms can be explained by the model.

**Table 2:** The results of stepwise multiple regression analysis of PHQ-9.

Variable selected	*B*	*SE*	*F*	*P*-value
Intercept	6.880	1.817	14.34	<0.001
Sex	2.002	0.683	8.58	0.0036
Neuroticism score (EPQ)	0.977	0.099	97.68	<0.001
Emotional abuse (CTQ)	0.296	0.122	5.85	0.0162
Active coping (SCSQ)	-0.195	0.058	11.16	<0.001
Use of social support (SSRS)	-0.379	0.191	3.95	0.0477
*R*^2^ = 0.4752				<0.001

*B, partial regression coefficient; SE, standard error. Dependent factor, PHQ-9 summary score. Twenty independent factors: age, sex (female = 1, male = 0), employment status (unemployed = 1, employed = 0), marital status (married = 1, unmarried = 0), living-alone (yes = 1, no = 0), presence of offspring (yes = 1, no = 0), comorbidity of physical disease (yes = 1, no = 0), first-degree relative with psychiatric disease (yes = 1, no = 0), first-degree relative with psychiatric disease (yes = 1, no = 0), SA, PA, EA, EN, PN of CTQ, EPQ neuroticism score, objective support, subjective support, the use of social support of SSRS, active coping, passive coping of SCSQ. CTQ, The Childhood Trauma Questionnaire; EPQ, Eysenck Personality Questionnaire; SSRS, the Social Support Rating Scale; SCSQ, Simplified Coping Style Questionnaire; PhQ-9, Patient Health Questionnaire-9.*

### Analysis of the SEM

**FIGURE 2:**
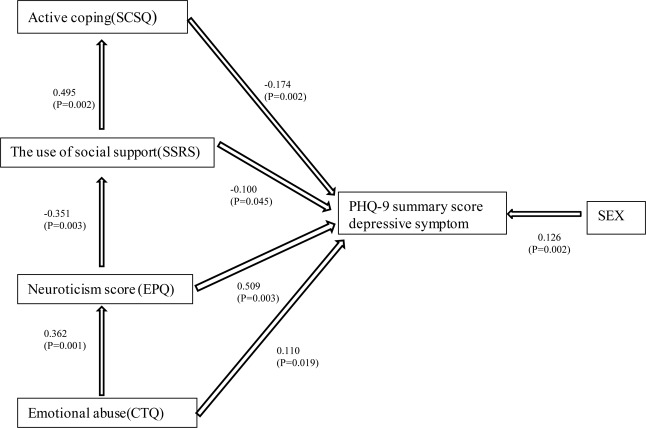
Covariance structure analysis in 312 participants. The results of the covariance structure analysis in the SEM with EA (CTQ), neuroticism (EPQ), the use of social support (SSRS), active coping (SCSQ), and depressive symptoms (PHQ-9) in 312 participants from the general adult population. Rectangles indicate the observed variables. The arrows with double lines indicate the statistically significant effects. The numbers beside the arrows show the standardized path coefficients (–1 to +1). CTQ, The Childhood Trauma Questionnaire; EPQ, Eysenck Personality Questionnaire; SSRS, The Social Support Rating Scale; SCSQ, Simplified Coping Style Questionnaire; PHQ-9, Patient Health Questionnaire-9.

Based on the hypothesis and the result of multiple regression analysis, a SEM was constructed to examine the relationship among all of the variables. The results of the path coefficients calculated by AMOS are shown in Figure 2. The goodness-of-fit indicators of the model were obtained with GFI = 0.944 and the coefficient of each path was substantially significant. The standardized direct path coefficient of predictors on depressive symptoms was: EA 0.110, *P* = 0.019, EPQR-S neuroticism 0.509, *P* = 0.003, the use of social support -0.100, *P* = 0.045, active coping -0.174, *P* = 0.002. The standardized indirect path coefficient of predictors on depressive symptoms was: EA 0.208, *P* = 0.001 (mediated by neuroticism, active coping, and use of social support), EPQR-S neuroticism 0.065, *P* = 0.002 (mediated by use of social support and active coping), the use of social support -0.086, *P* = 0.002 (mediated by active coping score). The result of SEM analysis for the model was in accordance with the multiple regression analysis (Table 2) and indicated that the data supported the theoretical model.

## Discussion

A main goal of this study was to examine the influence order and calculate the total effect of childhood abuse, neuroticism, social support, and coping style on depressive symptoms. The results from this study indicated that emotional childhood abuse, neuroticism, the use of social support, and active coping style affected depressive symptoms in turn and a total of 45.6% variability in depressive symptoms can be explained by them. In the constructed SEM, coefficient of each path was substantially significant.

As the results indicated, it is childhood EA that is related to depressive symptoms, rather than SA or PA. The result is consistent with earlier findings that EA is differentially associated with depression compared with other types of maltreatment (Gibb et al., 2003; Gibb and Abela, 2007; Calvete, 2014; Shapero et al., 2014; Ono K. et al., 2017). EA refers to the experience of being assaulted, rejected, humiliated, degraded, threatened, terrorized, isolated, or teased in childhood (Shapero et al., 2014). A theory has been proposed that compared with either physical or SA those who experience childhood EA may be more likely to develop negative self-associations and cognitive vulnerability to depression, since the negative evaluations and depressive cognitions are supplied directly to the child during emotionally abusive episodes (Rose and Abramson, 1992; van Harmelen et al., 2010). Antypa and Van der Does (2010) put forward that children experiencing other types of maltreatment (e.g., PA, SA) had to make their own attributions, which may allow more room for less global and more external attributions. Children who reported EA tended to develop a general negative attributional style, and it would contribute to the development of depression.

In this study, neuroticism is an important mediating factor between childhood EA and depressive symptoms. This is in accordance with previous studies that found neuroticism to be associated with childhood abuse in older person (Wilson et al., 2006) and a worse prognosis of depression (Bukh et al., 2016). It is possible that specific forms of childhood abuse are associated with particular personality traits, and neuroticism was especially associated with EA (Li et al., 2014b). Another study documented that childhood EA have possible relation with poor impulse control (Rademaker et al., 2008). Individuals used to be in an emotional abusive environment would use psychological defense mechanism excessively to adjust the contradiction between the reality and internal needs, and this would gradually cause personality deviation (Li et al., 2014b). Most previous researches have focused on childhood PA and SA and their impact on personality (Pitzer and Fingerman, 2010). For example, sexual and PA have strong correlations with antisocial personality disorder and borderline personality disorder (Huang et al., 2012; Zhang et al., 2012). However, this study raises attention to EA and its impact on personality characteristic.

Higher social support plays a protective role against the development of depression (Boen et al., 2012), and it adds a feeling of connectedness. This is in line with the psychosocial theory (Aktan, 2012; Jeong et al., 2013). Our study reported that social support buffers the impact of childhood abuse on depressive symptoms, which is consistent with previous researches (Muzik et al., 2017). However, the research indicated that the impact of support utilization is greater than that of objective support and subjective support. The support will be most effectual when it is sufficiently utilized, suggesting taking effective interventions to increase the ability of utilizing support may contribute mostly to improving depressive symptoms (Shao et al., 2018). Social support is a relatively low-cost intervention. Further investigation of effective and efficient methods to deliver social support interventions and improve the ability of utilizing social support would be worthwhile (Yoo et al., 2017). The pervasive application of social media has changed communication habits among patients and provides the opportunity to access social support online, which will accordingly increase the chance of social support utility.

Social support and coping style are strongly interdependent (Söllner et al., 1999). They are considered as factors that increase resiliency to mental health issues. Positive coping mechanisms such as seeking social support are positively associated with resiliency (Howe et al., 2012; Thompson et al., 2016). The buffering effects of social support on health outcomes are often mediated by the coping behaviors. Higher level of social support may enhance the subject’s fighting spirit and adequate use of social support contributes to more active coping style (Aymanns et al., 1995; Söllner et al., 1999). The regression model (Table 2) and structural analysis (Figure 2) indicated that the interaction between use of social support and active coping style are significantly and negatively correlated with the severity of depressive symptoms. This was in line with the result of a survey conducted among pregnant women after the Lushan earthquake in Ya’an, China, which suggested that the effect of social support on depression was mediated by active coping (Ren et al., 2015). This may be due to the fact that social environment may influence the choice of a specific coping strategy and the effectiveness of the strategy used. People unsatisfied with social support or with lower use of social support tended to utilize avoidant coping style when they were in depressed mood (Schwarzer and Weiner, 1991; Rudnicki et al., 2001).

There are some strengths and limitations of this study. Major strength is that this study covers relatively comprehensive and systematic mediating factors between childhood maltreatment and depressive symptoms, including personality, social support, and coping strategies. Several limitations need to be mentioned as well. First of all, we need to be cautious in the interpretation of the causal inference and directions between study variables. The model was built on the basis of a cross-sectional study rather than a prospective longitudinal study. Secondly, the recall bias should be considered, as the childhood maltreatment was investigated retrospectively using self-report questionnaires.

## Conclusion

By using SEM in the population covering general adults, depressed patients, bipolar disorder patients, and high risk population for depression, our hypothesis was verified that childhood EA affects depressive symptoms through neuroticism, the use of social support, and active coping style in turn. These analyses presented a tentative developmental model for the etiology of major depression, and a large-scale prospective study will be necessary to test and verify.

## Ethics Statement

Ethical approval was obtained from the Ethics Review Committee of Beijing Anding Hospital, Capital Medical University (201467). All patients participated on a voluntary basis and gave their written informed consent before data collection.

## Author Contributions

JZ performed the statistical analyses and wrote the manuscript. LF supervised the project and assisted in writing the manuscript. GW initiated and designed the study and wrote the protocol. CH collected the data. LX and CP reviewed the manuscript. All authors read and approved the final manuscript.

## Conflict of Interest Statement

The authors declare that the research was conducted in the absence of any commercial or financial relationships that could be construed as a potential conflict of interest.

## Abbreviations

CTQ-SF: childhood trauma questionnaire
DSM-IV: diagnostic and statistical manual of mental disorders fourth edition
EA: emotional abuse
EN: emotional neglect
EPQR-S: eysenck personality questionnaire
FMDR: family history of mental illness in first-degree relatives
GFI: goodness of fit index
MDD: major depressive disorders
MINI: mini international neuropsychiatric interview
Ns: not significant
PA: physical abuse
PHQ-9: patient health questionnaire-9
PN: physical neglect
SA: sexual abuse
SCSQ: simplified coping style questionnaire
SD: standard deviation
SEM: structural equation modeling
SMDR: family history of mental illness in second-degree relatives
